# From promise to practice: digital health through the lens of neonatal care

**DOI:** 10.1016/j.lana.2026.101600

**Published:** 2026-08-28

**Authors:** Gabriel Fernando Todeschi Variane, Danieli Mayumi Kimura Leandro, Marcelo Jenne Mimica, Topun Austin, Valerie Y. Chock, Alexa Craig, Khorshid Mohammad, Krisa Page Van Meurs

**Affiliations:** a Division of Neonatology, Department of Pediatrics, Irmandade da Santa Casa de Misericórdia de São Paulo, São Paulo, Brazil; b Protecting Brains & Saving Futures, Clinical Research Department, São Paulo, Brazil; c Santa Casa de São Paulo School of Medicine, São Paulo, Brazil; d Neonatal Intensive Care Unit, Cambridge University Hospitals NHS Foundation Trust, Cambridge, UK; e Division of Neonatal and Developmental Medicine, Stanford University School of Medicine and Lucile Packard Children's Hospital Stanford, Palo Alto, CA, United States; f Department of Pediatrics, Barbara Bush Children's Hospital at MaineHealth, MaineHealth Neurology Scarborough, Scarborough, ME, USA; g Pediatrics, Tufts University School of Medicine, Boston, MA, USA; h Pediatrics, Alberta Children's Hospital, Calgary, Canada

**Keywords:** Digital health, Telehealth, Aritificial intelligence, Neonatal neurology, Neonatology, Neurocritical care

## Abstract

Digital health is no longer a future prospect, it is already embedded in clinical care. Yet its impact remains inconsistent. Bridging this gap between technological capability and clinical value is now a central challenge for health systems. The field of neonatology offers a critical test case, combining extreme physiological vulnerability, high-density multimodal data, and time-sensitive decision-making, where early clinical events and interventions shape outcomes across the lifespan. In this setting, large-scale implementation of remote multi-modality monitoring and tele–neurocritical care across national networks demonstrate that digitally enabled models can extend expertise and support more consistent high-quality care. In this Viewpoint, we argue for shifting from isolated technologies to integrated systems, where continuous, multimodal data enable actionable bedside decisions through aligned layers of intelligence, clinical interfaces, and enabling infrastructure. We further examine the challenges that accompany this transition and the limited inclusion of long-term outcomes, particularly in vulnerable populations. We outline the governance conditions required for responsible and scalable implementation. The future of digital health will not be determined by technological sophistication alone, but by its ability to deliver equitable, durable improvements in patient outcomes. Digital innovation when embedded within structured systems in the neonatal environment can move from promise to practice.

## Introduction

Digital health has moved well beyond proof of concept. Artificial intelligence (AI), continuous biosensing, and networked models of care are increasingly gaining traction in clinical workflows across multiple health systems, influencing how diagnoses are considered, risks are anticipated, and decisions are made.^1^^,^^2^ Despite rapid technological progress, the real-world impact of digital health remains inconsistent. Many innovations stall at the pilot stage, fail to scale, or deliver results that are disconnected from meaningful patient outcomes. This gap between technological promise and clinical practice represents one of the central challenges facing contemporary health systems.^2^

Critical care offers a uniquely instructive lens through which to examine the challenge of digital health. Neonatal intensive care units (NICUs) combine extreme physiological vulnerability, high-density multimodal data, and time-critical decision-making, where early clinical events and interventions can influence health trajectories across the entire lifespan.^3^^,^^4^ These characteristics make neonatology both an ideal high-acuity environment for digital innovation implementation and a sensitive test of whether digital health systems truly improve care and outcomes or whether it merely increases clinical complexity.

Importantly, neonatal digital health has moved beyond experimentation. Large-scale implementations of remote monitoring, tele–neurocritical care, and standardized multimodal neuromonitoring have shown that digitally enabled neonatal care can be deployed across regional and national networks, including in middle-income countries, with reported clinical and organizational benefits.^5^^,^^6^ A recent publication by Variane et al. describes the implementation of a nationwide digital health network providing remote neuromonitoring to more than 11,000 infants, along with telehealth consultations and longitudinal education in more than 70 centers across Brazil.^5^ These reports challenge the assumption that advanced digital health is restricted to highly resourced settings and suggest that neonatology and neonatal neurocritical care serve as a central field for understanding how digital health can be implemented responsibly at scale in a wide variety of settings.

Continuous biosensing and AI-assisted interpretation have been described to improve early detection of clinical deterioration, support prognostication, and expand access to specialist expertise.^7^^,^^8^ However, these advances also introduce critical risks. Algorithmic and automation biases, weak governance, and limited assessment of long-term outcomes may compromise potential gains, particularly in populations already experiencing structural inequities.^9^ In neonatal care, where decisions made in the first days of life can shape neurodevelopmental and functional outcomes for decades, these risks carry exceptional ethical implications.

In this Viewpoint, we argue that the success of digital health depends not only on technological novelty but also on implementation models that integrate continuous data, clinical governance, cost-effectiveness, and equity-oriented system design. Using neonatal care as a model, we examine how emerging technologies can move from data generation to decision-making, identify conditions under which digital systems meaningfully improve outcomes, and highlight the governance principles required to ensure that digital health strengthens, rather than fragments, health systems across the life course.

## Neonatal care as a model for digital health systems

Neonatal care represents one of the most complex and data-intensive environments in contemporary medicine and therefore serves as a powerful model for evaluating digital health systems. It continuously generates high-frequency, multimodal data streams, including physiological waveforms, neurophysiological monitoring data, advanced imaging, multiple ventilatory parameters, laboratory biomarkers, and increasingly, genomic and other omics data, while clinical decisions must often be made within narrow therapeutic windows.^10^, ^11^, ^12^ This convergence of data density, physiological instability, and time-sensitive interventions creates an ecosystem in which both the capabilities and limitations of digital technologies become rapidly apparent.

Beyond technical complexity, neonatal care has profound ethical and societal consequences. Decisions made in the first hours and days of life can shape neurodevelopmental trajectories, functional capacity, and health across the lifespan.^4^ This amplifies both the potential benefits and the risks of digital decision-support systems. Tools that enhance early detection of clinical deterioration, support standardized neurological assessment, and facilitate timely access to specialist expertise may generate durable benefits, while inadequately validated or poorly governed systems risk compounding harm at a particularly vulnerable stage of life.^13^

Until relatively recently, healthcare professionals assessed patients by observing monitors and vital signs in isolation. Heart rate, blood pressure, oxygen saturation, and continuous brain monitoring data, were interpreted separately, often relying heavily on the experience and moment-to-moment judgment of the medical provider. This approach inherently limits the ability to capture the complex, dynamic interplay between physiological systems. Today we are witnessing a paradigm shift: data integration, advanced analytics, and AI are enabling a more holistic and continuous understanding of patient physiology. Instead of fragmented signals, it has become possible to interpret patterns, trends, and interactions across multiple data streams in real time, enhancing early detection of deterioration, improving decision-making, and moving care closer to a truly precision-based, proactive model.^14^ Experience with automated seizure detection, early warning systems, and imaging analysis demonstrates both the promise of multimodal AI-assisted interpretation and the methodological challenges related to dataset quality, overfitting, and generalizability.^15^^,^^16^ These experiences provide a conceptual and practical foundation for the integration of more advanced digital health approaches, including AI-assisted interpretation and networked models of care.^17^^,^^18^

Lessons derived from neonatal digital health may extend to other high-acuity fields such as adult and pediatric critical care, emergency medicine, maternal–fetal medicine, and a variety of pediatric chronic diseases, as these domains share challenges such as clinical instability, complex data streams, and long-term impact.

## From data to decisions: the digital care stack

**Fig. 1:**
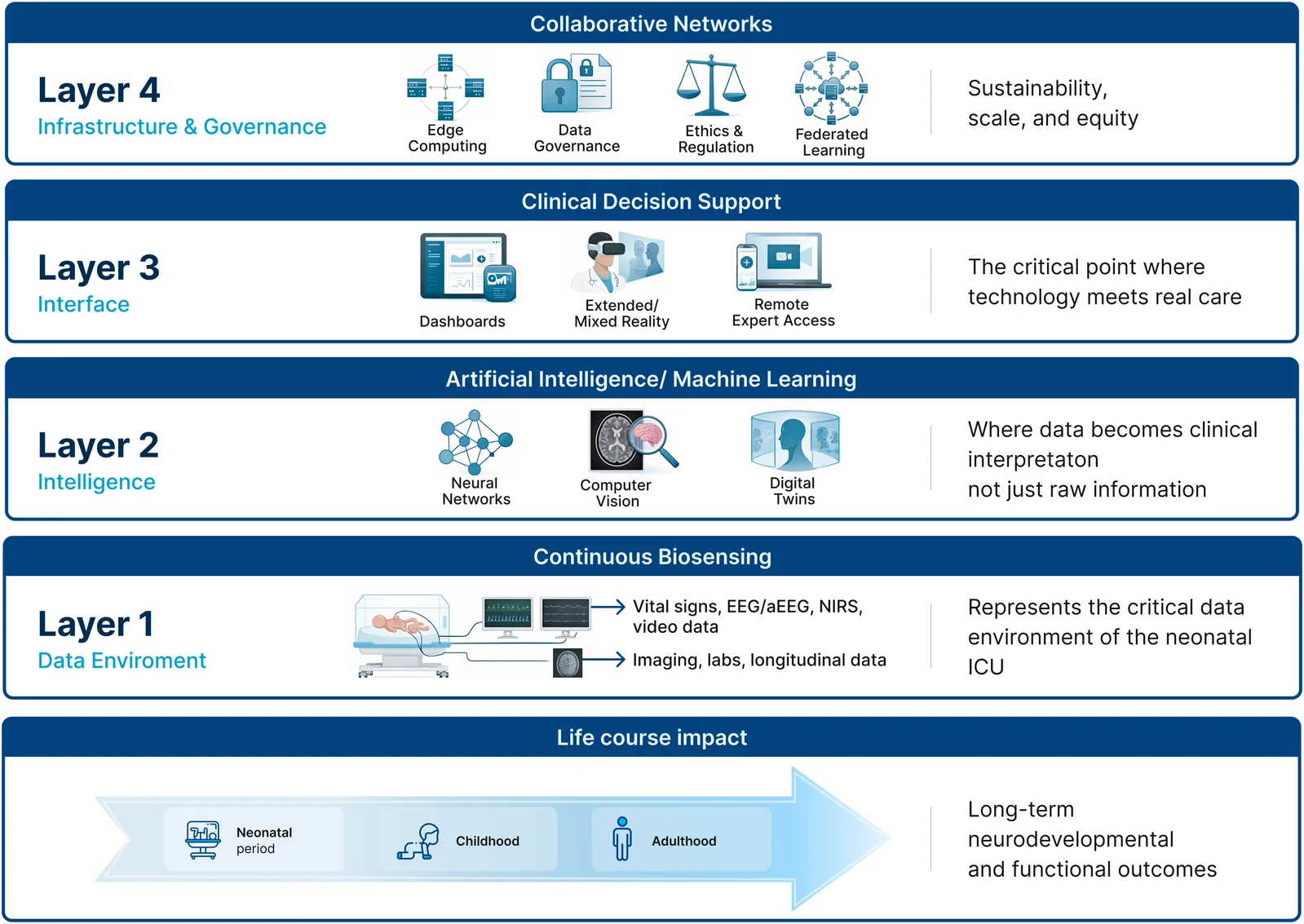
Neonatal care as a sentinel model for digital health: from data to decisions across the life course. Neonatal intensive care concentrates continuous, multimodal data and time-critical decision-making, providing a high-stakes environment to evaluate digital health systems. A layered digital care stack (from data acquisition to intelligent interfaces and governance) illustrates how technology can move from data generation to clinical decision-making, with implications that extend across the life course and are transferable to other health systems.

**Fig. 2:**
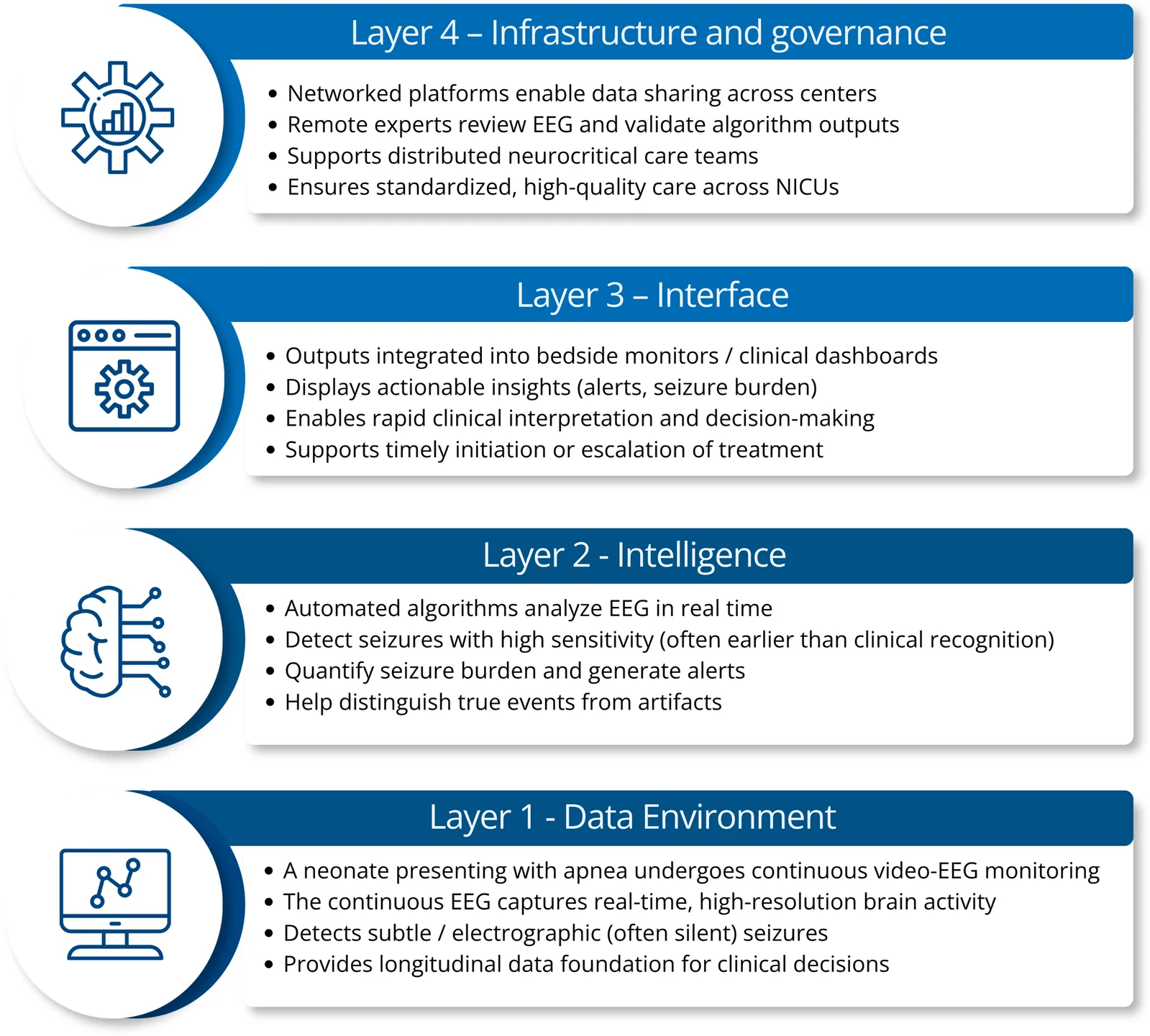
From signal to care: A neonatal seizure case across data, intelligence, interface, and infrastructure layers.

Digital transformation in health care can be understood as a layered care stack spanning data generation, computational intelligence, clinical interfaces, and enabling infrastructure, through which raw signals are translated into actionable and scalable clinical decisions. This framework is illustrated in Fig. 1, and its potential application in a clinical scenario is shown in Fig. 2.

### Data environment layer—continuous and multimodal sensing

The foundation of digital health transformation lies in the capacity to acquire continuous, high-resolution, and multimodal clinical data. Electronic health records (EHRs) provide both structured and unstructured information that can complement continuous physiological monitoring. Advances in large language models are increasingly enabling the extraction, synthesis, and contextualization of clinical narratives alongside imaging and laboratory data.^19^^,^^20^ At the same time, the Internet of Medical Things (IoMT), bedside monitoring systems, and non-invasive biosensors enable continuous longitudinal capture of physiological and neurophysiological signals. These complementary data streams create a richer and more dynamic representation of patients’ conditions, enabling earlier recognition of clinical deterioration, more informed decision-making, and timely detection of evolving brain injury during neonatal care.^10^^,^^21^

Beyond conventional monitoring, the integration of multi-omics data offers the potential to complement physiological signals with molecular insights into disease susceptibility and treatment response. Although the ‘omics’ approaches remain largely confined to research and selected clinical indications in neonatology, declining costs and improving analytical frameworks suggest an expanding role in risk stratification and precision medicine.^22^ Crucially, the clinical value of these data depends not on volume alone, but on their integration into interpretable and clinically actionable frameworks.

### Intelligence layer—making sense of complexity

As data volume and complexity increasingly exceed human cognitive capacity, AI and machine learning have emerged as critical tools for interpretation and synthesis. In neonatal and pediatric care, AI-driven models have been applied to early warning systems for life-threatening complications such as sepsis, hypoxic-ischemic encephalopathy, necrotizing enterocolitis, seizure detection, imaging interpretation, and outcome prediction, often demonstrating improved sensitivity and timeliness compared with traditional approaches.^15^^,^^23^^,^^24^ Among these applications, automated seizure detection has emerged as one of the most mature examples of successful clinical translation, with machine-learning algorithms demonstrating robust performance in identifying neonatal seizures from continuous EEG recordings.^25^

Computer vision has further expanded these capabilities through automated analysis of medical images and video data, supporting standardized assessments and reducing inter-observer variability.^26^ Digital twins, defined as dynamic computational representations integrating multimodal longitudinal data to simulate disease trajectories or therapeutic responses, are emerging as a potential framework for personalized decision support.^27^ While these applications have shown considerable promise, clinical adoption remains at varying stages of maturity. As they move toward broader implementation, their greatest value is likely to come from augmenting, rather than replacing clinical reasoning, reinforcing the need for human oversight in high-risk environments such as neonatal care.

Beyond prediction and automation, AI-based digital health platforms can offer an opportunity for scientific discovery. Large multimodal neonatal datasets have the potential to uncover previously unrecognized physiological relationships, disease trajectories, and treatment-response patterns. Such data-driven hypothesis generation may ultimately accelerate mechanistic research and lead to new treatments with improved outcomes.^28^^,^^29^

### Interface layer—translating intelligence into care

The clinical impact of digital intelligence is determined by how effectively insights are delivered at the point of care. Clinical decision-support systems that integrate multimodal data into concise, context-aware outputs have shown potential to improve adherence to evidence-based protocols and reduce unwarranted variability in clinical practice.^5^ In routine care, these capabilities are increasingly embedded within electronic medical records and bedside platforms, directly informing clinical actions. Examples include sepsis risk calculators guiding decisions before ordering blood cultures or initiating antibiotics, bilirubin nomograms supporting phototherapy initiation, and ventricular index charts integrated into cranial ultrasound reporting to standardize the assessment of ventriculomegaly.

Extended reality technologies, including augmented, mixed, and immersive virtual reality, represent an emerging interface between digital intelligence and bedside care. In the education of health providers and in selected clinical applications, these tools have enabled improvements in procedural guidance, skill acquisition, and remote expert support.^30^, ^31^, ^32^ In neonatal care, early pilot experiences suggest that immersive interfaces may support standardized neurological assessment and facilitate access to subspecialty expertise, particularly in settings with limited local resources.^33^ The success of such systems depends on usability, workflow integration, and clinician trust.

### Infrastructure layer—enabling scale

Scalable digital health systems require robust computational and governance infrastructure. Edge computing, a distributed information technology architecture that processes data near the source, enables real-time processing of high-frequency physiological data close to the point of care, reducing latency and reliance on centralized cloud resources. Federated learning, a machine learning approach where a model is trained across multiple institutions or devices without sharing the underlying raw data, further supports scalability by enabling collaborative model development across institutions without transferring raw patient data, addressing privacy, regulatory, and data-ownership concerns.^34^^,^^35^ These approaches are particularly relevant for neonatal networks spanning diverse healthcare systems.

Quantum computing, an emerging computational approach with the potential to analyze highly complex biomedical datasets beyond the capabilities of conventional computing, represents a more distant horizon. Although still largely experimental, its potential to process complex, high-dimensional biomedical data has generated interest in applications such as genomics and multimodal data integration.^36^ At present, quantum approaches remain conceptual in clinical care and should be viewed as long-term research directions rather than near-term solutions.

## Evidence from successful implementation: moving beyond pilots

While the digital care stack provides a conceptual architecture, its clinical relevance ultimately depends on real-world implementation within structured care pathways. Over the past decade, digital health in neonatal care has progressed from isolated pilot projects to structured, system-level implementation. Reported experiences indicate that digitally enabled models can extend specialist expertise beyond tertiary centers and contribute to more consistent clinical decision-making across hospital networks.^5^^,^^37^, ^38^, ^39^, ^40^ These initiatives demonstrate that scale and sustainability are achievable when digital tools are embedded within defined clinical pathways.

A central driver of implementation success has been the integration of continuous monitoring within standardized neurocritical care protocols and pathways. The increasing use of continuous electroencephalography (EEG), near-infrared spectroscopy (NIRS), multimodal monitoring, and protocolized neurological assessment has shown that complex physiological data can be translated into actionable bedside information.^11^^,^^41^ Importantly, implementation studies have taught us that impact depends less on technology itself and more on governance, training, and workflow integration.^41^^,^^42^ Programs that invest in education, shared protocols, and multidisciplinary collaboration report better adherence to evidence-based practices and stronger alignment between bedside teams and remote experts.^43^, ^44^, ^45^, ^46^

Experiences from diverse healthcare systems also challenge the assumption that advanced digital neonatal care is restricted to highly resourced settings. Network-based approaches, when adapted to local infrastructure and workforce capacity, can support quality improvement across diverse environments.^6^^,^^18^ In India, implementation of the Smart-NICU model across 18 neonatal intensive care units, encompassing 214 beds and caring for over 1600 newborns during its first 18 months, demonstrated the feasibility of combining low-cost digital technologies, remote specialist support, and structured training to expand access to neonatal critical care, including in rural settings.^47^ These findings position neonatal digital health as a practical strategy for strengthening systems through scalable, network-based models that improve access, consistency of care, and workforce capacity, not merely enhancing individual patient management.

## Risks, blind spots, and conditions for success

As digital health becomes increasingly embedded in intensive care, attention must shift from technical feasibility to the conditions required to sustain clinical benefit over time. One of the most frequently cited concerns is algorithmic bias. Models trained on limited or homogeneous datasets may underperform when applied to diverse populations, constraining generalizability across regions and health systems and potentially reinforcing existing inequities if deployed without appropriate safeguards.^9^^,^^13^ Fortunately, this risk is now widely recognized, and contemporary implementation efforts increasingly prioritize dataset diversity, external validation, and continuous performance monitoring as core design principles rather than post hoc adjustments.^48^, ^49^, ^50^

Automation bias represents a related but addressable challenge. As decision-support systems demonstrate improved accuracy and efficiency, clinicians may over-rely on algorithmic outputs.^9^^,^^13^^,^^49^ In neonatal care, where early decisions can shape neurodevelopmental trajectories across the life course, digital tools must be intentionally designed to support, rather than replace, clinical reasoning. Transparency, interpretability, explainability, and clearly defined roles for human oversight are therefore essential enablers of trust and safe adoption, rather than barriers to innovation.^1^^,^^50^ These risks do not argue against digital health, but rather clarify the conditions under which it can be responsibly scaled.

Early digital health experiences have appropriately focused on short-term outcomes, including diagnostic accuracy, workflow efficiency, and in-hospital endpoints. This emphasis reflects a natural and necessary first phase of innovation, in which feasibility, safety, and immediate clinical relevance must be established.^50^ As digital care matures, the logical next step is the systematic evaluation of long-term neurodevelopmental, functional, and quality-of-life outcomes. In neonatology, this progression is particularly compelling, as early-life interventions can influence health trajectories across an individual's lifespan.

Importantly, the same digital infrastructures that enable continuous monitoring in the NICU may be leveraged to support longitudinal follow-up. Integrated data platforms, remote assessment tools, and standardized outcome frameworks could provide scalable and resource-efficient mechanisms to extend evaluation beyond hospital discharge.^51^^,^^52^ Embedding long-term outcome assessment within digital neonatal care pathways therefore represents a logical and natural extension of current practice by demonstrating sustained, patient-centered value. Mobile applications enabling parents to capture infants’ movements and behaviors in the home environment offer a powerful alternative to in-person neurodevelopmental assessments, particularly where geographic and financial barriers limit access to follow-up. Further development of harmonized longitudinal registries and interoperable digital follow-up platforms may contribute to mitigating methodological barriers such as attrition and heterogeneity in outcome measurement.

Technologies in healthcare can have a significant environmental impact, due to energy consumption, electronic waste, and the infrastructure required to sustain continuous data flows. Data centers, for instance, demand substantial electricity and cooling resources, while the rapid turnover of medical and digital equipment contributes to e-waste challenges. However, these same technologies also offer powerful opportunities to offset their environmental impact. By enabling telemedicine, remote monitoring, and more efficient care pathways, digital health can reduce patient travel, optimize resource utilization, decrease unnecessary hospitalizations, and minimize waste from redundant testing and procedures. Thus, while digitalization introduces new environmental burdens, it also provides critical tools to potentially redesign healthcare in a way that is ultimately more efficient and sustainable.

Finally, accumulated experience consistently shows that success depends less on technology itself than on sociotechnical integration.^53^^,^^54^ These lessons suggest that the path forward lies not only in accelerating technological innovation, but also in consolidating implementation models that translate digital capability into meaningful and lasting benefit for newborns, families, and health systems.

## Governance, equity, and a global perspective

**Fig. 3:**
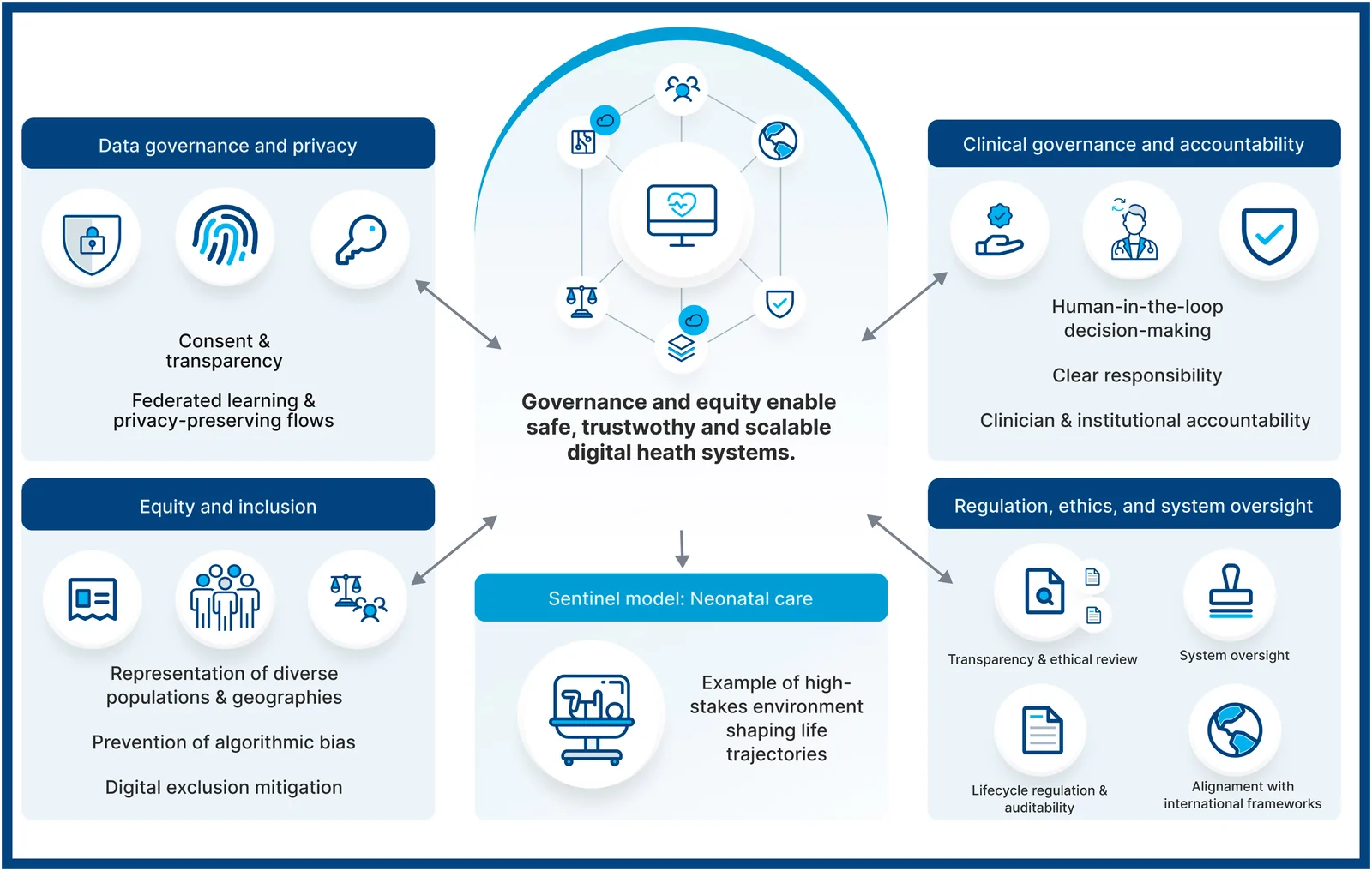
Governance and equity as enabling foundations for digital health. Integrated governance frameworks addressing data stewardship, clinical accountability, equity, and regulatory oversight support safe scale-up of digital health systems. Using neonatal care as a sentinel model, the figure illustrates how equity-oriented and transparent governance enables bias mitigation, trust, and sustainable implementation across diverse populations and health-system contexts.

Digital health innovation is inherently global and increasingly embedded in routine clinical care. However, the long-term storage, curation, and sharing of high-resolution data may limit interoperability and slow the scalability of these solutions. Differences in data governance, cybersecurity requirements, and institutional policies may hinder real-time data sharing and cross-network collaboration. Nonetheless, these should not be insurmountable barriers to progress, but essential conditions to be addressed through coordinated policy, robust infrastructure, and responsible system design. In high-risk environments such as neonatal intensive care, governance frameworks are evolving in parallel to support safe and scalable implementation. Interoperable architectures and federated learning approaches may help address these constraints while preserving privacy and local data ownership. Rather than functioning as constraints, ethical, regulatory, and operational oversight structures are progressively becoming enablers of trustworthy digital systems (Fig. 3).^1^^,^^51^

The evolution of AI-enabled clinical tools has been accompanied by parallel advances in lifecycle oversight models, reflecting a growing alignment between innovation and regulation. Rather than relying solely on static pre-approval paradigms, contemporary regulatory approaches increasingly embrace adaptive monitoring, post-deployment evaluation, and proportional risk-based oversight as mechanisms to sustain both safety and progress.^55^ These evolving frameworks are particularly well-suited to neonatal care, where continuous data streams and longitudinal outcomes create unique opportunities for ongoing learning, refinement, and performance optimization.

Algorithmic systems, when embedded within structured monitoring environments, can be continuously audited and refined. Subgroup performance analysis, transparent validation reporting, and periodic recalibration offer mechanisms to mitigate dataset shift and maintain performance across diverse populations.^1^^,^^51^ Far from representing insurmountable barriers, these processes exemplify how digital infrastructures can support learning health systems capable of iterative improvement.

Advances in data architecture further expand the horizon of collaborative innovation. Federated learning and distributed analytic approaches enable institutions to contribute to model development while preserving patient privacy and local governance.^56^ Aligned with established health data governance principles, such architectures create pathways for large-scale validation without compromising accountability.^57^ In neonatal networks, where case volumes for specific conditions may be limited at individual centers, these collaborative models are especially promising.

Equity, in this context, becomes both an ethical imperative and a design principle. Transparent model development, inclusive data representation, and preservation of clinician and family decision-making authority reinforce trust in digitally supported care.^55^ Human-in-the-loop frameworks ensure that AI augments, rather than displaces, clinical judgment, maintaining the relational core of neonatal medicine while enhancing precision.^51^

Economic sustainability is not separate from innovation but integral to it. Digitally enabled network models can extend specialist expertise, reduce unwarranted variation, and support earlier intervention, benefits that, when evaluated through health economic frameworks, may justify investment in scalable digital infrastructure.^58^^,^^59^

As evidence accumulates, digital care may increasingly be understood not as an incremental expense, but as foundational infrastructure for resilient, learning health systems. When innovation is coupled with transparent oversight and collaborative data architectures, digital systems can be evaluated, refined, and scaled with greater confidence and accountability.

## Conclusion

Emerging health technologies are no longer speculative tools of future medicine, they are actively reshaping clinical care today. Experiences from neonatal medicine demonstrate that, when embedded within structured care pathways, digital systems can enhance clinical precision, extend specialist expertise, and improve continuity of care across geographically diverse settings. Large-scale digital health initiatives provide evidence that meaningful transformation is potentially achievable not only in highly resourced environments, but also within low-to middle-income health systems.

Neonatal care reveals that the lasting value of digital health lies in its capacity to build durable clinical infrastructure and reorganize care around data-informed, accountable decision-making. In this context, AI-enabled support and continuous multimodal monitoring have the potential to strengthen clinical judgment, reduce variability, and facilitate earlier intervention while preserving the relational core of medicine.

The ultimate measure of digital health will rest not on technological sophistication alone, but on its ability to advance resilient and equitable health systems and improve patient outcomes. When innovation is anchored in evidence, reinforced by adaptive governance, and sustained through rigorous real-world implementation, digital transformation moves beyond aspiration to become an achievable standard of care.

## Contributors

All authors were responsible for conceptualization, data curation, supervision, validation, visualization, writing, and revising the article.

## Declaration of interests

The authors declare no conflict of interest.
